# Discrimination of genetic and geographical groups of grape varieties (*Vitis vinifera* L.) based on their volatile organic compounds

**DOI:** 10.3389/fpls.2022.942148

**Published:** 2022-10-20

**Authors:** Iva Šikuten, Petra Štambuk, Ivana Tomaz, Cecile Marchal, Jasminka Karoglan Kontić, Thierry Lacombe, Edi Maletić, Darko Preiner

**Affiliations:** ^1^Department of Viticulture and Enology, Faculty of Agriculture, University of Zagreb, Zagreb, Croatia; ^2^Centre of Excellence for Biodiversity and Molecular Plant Breeding, Faculty of Agriculture, University of Zagreb, Zagreb, Croatia; ^3^Grapevine Biological Resources Center, INRAE, Unité Experimental Domaine de Vassal, University of Montpellier, Marseillan, France; ^4^AGAP, University of Montpellier CIRAD, Institut Agro, Montpellier, France

**Keywords:** volatile organic compounds, grapevine varieties, GEN-GEO groups, discrimination, volatile profiles

## Abstract

Grape volatile organic compounds (VOCs) play an important role in the winemaking industry due to their contribution to wine sensory characteristics. Another important role in the winemaking industry have the grapevine varieties used in specific regions or countries for wine production. Due to the high variability of grapevine germplasm, grapevine varieties are as classified based on their genetic and geographical origin into genetic-geographic groups (GEN-GEO). The aim of this research was to investigate VOCs in 50 red grapevine varieties belonging to different GEN-GEO groups. The study included varieties from groups C2 (Italy and France), C7 (Croatia), and C8 (Spain and Portugal). The analysis of VOCs was performed by SPME-Arrow-GC/MS directly from grape skins. The analyzed VOCs included aldehydes, ketones, acids, alcohols, monoterpenes, and sesquiterpenes. The most abundant VOCs were aldehydes and alcohols, while the most numerous were sesquiterpenes. The most abundant compounds, aldehydes and alcohols, were found to be (*E*)-2-hexenal, hexenal, (*E*)-2-hexen-1-ol, and 1-hexanol. Using discriminant analysis, the GEN-GEO groups were separated based on their volatile profile. Some of the individual compounds contributing to the discrimination were found in relatively small amounts, such as benzoic acid, (*E,E*)-2,4-hexadienal, 4-pentenal, and nonanoic acid. The groups were also discriminated by their overall volatile profile: group C2 was characterized by a higher content of aldehydes and alcohols, and group C8 was characterized by a higher content of sesquiterpenes and acids. Group C7 was characterized by all low amount of all classes of VOCs.

## Introduction

According to OIV (2017), there are 6,000 grapevine varieties in the world, which were developed during the long domestication history of grapevine by the combined actions of selection, breeding, admixture, and migration (Bacilieri et al., 2013). Traditionally, grapevine varieties are classified based on their usage in wine and table grapevine varieties. However, the development of biochemical and molecular markers enabled the assessment of the genetic diversity and structure of grapevine varieties. Using different molecular markers and structure analysis, many authors have classified grapevine varieties into different genetic groups (Aradhya et al., 2003; Arroyo-Garcia et al., 2006; Bacilieri et al., 2013; Laucou et al., 2018), which overlap with certain geographic areas and confirm the accepted classification of Negrul et al. (1946). The GEN-GEO groups of varieties included in this paper are based on the work of Laucou et al. (2018). The authors divided a large sample of 783 grapevine varieties into eight GEN-GEO groups using single nucleotide polymorphism (SNP) markers and structure analysis. The groups are C1 (cultivars from Western and Central Europe, and Iberian Peninsula), C2 (similar to group C1 with the addition of wine cultivars from the Italian peninsula), C3 (wine and table cultivars from the Iberian Peninsula), C4 (table cultivars from Western Europe), C5 (table cultivars from Eastern regions), C6 (wine cultivars from Eastern Mediterranean and Caucasus regions), C7 (cultivars from the Balkan region), C8 (mainly Iberian cultivars, as well as cultivars from Western Europe, the Balkan region, and the Italian peninsula). This classification was used in our previous work (Šikuten et al., 2021b), where we investigated polyphenolic profiles of GEN-GEO groups and used these profiles in discriminant analysis.

In the last 40 years, almost 1,000 volatile compounds have been identified in wine, with a content ranging from μg/L up to mg/L (Pons et al., 2017). VOCs are secondary metabolites responsible for grape and wine sensory properties. They can come from several sources: directly from grape berry, from alcoholic fermentation through yeast and bacterial metabolism, or from aging (Francis and Newton, 2005). VOCs that come directly from grapes are the product of the grapes’ own metabolism, therefore influenced by grape variety, climate conditions, and viticultural practices (Bretón et al., 2020). The main groups of volatile compounds in grapes are terpenoids (monoterpenes, sesquiterpenes), C_13_-norisoprenoids, alcohols, carbonyls, and methoxypyrazines. Furthermore, all these compounds can be found in free form, as volatile molecules, or in glycosidically bound form, as non-volatile molecules.

Monoterpenes are a class of compounds that give rise to Muscat’s characteristic floral aroma. In grape berries, they can be found both in skins and pulp with different distributions, depending on the compound (Luan and Wust, 2002), and can be found as free volatile compounds or as non-volatile glycosidically bound compounds (Ilc et al., 2016). Most of the wine monoterpenes contribute toward floral and citrusy notes (Ilc et al., 2016). The main representatives and most odoriferous are monoterpene alcohols, notably linalool, citronellol, *α*-terpineol, nerol, geraniol, and hotrienol (Gonzalez-Barreiro et al., 2015). Sesquiterpenes in grapes and wines have received less attention due to their lower volatility and higher detection threshold (Lin et al., 2019). In a recent review by Li et al. (2020), the authors extensively summarized the presence and impact of 97 sesquiterpenes in grapes and wines. Despite the numerous compounds present in grapes, the most significant sesquiterpene is rotundone, a compound responsible for the peppery aroma of Australian shiraz wines (Wood et al., 2008). Norisoprenoids are a diverse group of widespread compounds derived from the oxidative breakdown of carotenoids (Rienth et al., 2021). Similar to monoterpenes, their aroma is mostly described as floral or fruity, and the majority of compounds can be found as non-volatile glycosides (Ilc et al., 2016; Lin et al., 2019). The most important norisoprenoids are *β*-damascenone, *β*-ionone, vitispirane, and TDN (1,1,6-dimethyl-1,2-dihydronaftalene; Gonzalez-Barreiro et al., 2015). Methoxypyrazines are a class of nitrogenated heterocyclic compounds found in many plants, contributing to aromas described as herbaceous, green, vegetal, and earthy (Ilc et al., 2016; Rienth et al., 2021). These compounds are characterized by an extremely low odor detection threshold, and their excessive levels may result in unacceptable green and unripe aromas that negatively affect wine quality (Lei et al., 2018). C_6_ and C_9_ alcohols and aldehydes are products of the lipoxygenase pathway and have the characteristic green aroma that can be a negative contributor to wine aroma (Ilc et al., 2016; Lin et al., 2019). However, the levels of these compounds in wines are mainly modulated by the winemaking process. This happens when the more odorous C_6_ aldehydes are reduced to less odorous C_6_ alcohols by yeast activity during alcoholic fermentation (Rienth et al., 2021).

There is a lot of research regarding the aromatic profiles of grape varieties, the influence of climate conditions on the accumulation of VOCs, and the effect of winemaking practices on the sensory properties of wine. However, only recently has research started to elucidate the genetic mechanism behind the biosynthesis and metabolism of grape VOCs (Lin et al., 2019). The grape VOCs and their precursors originate from multiple biosynthetic pathways and can undergo enzyme-catalyzed modifications and spontaneous chemical transformations (Dunlevy et al., 2009; Lin et al., 2019), thus making the research on the biosynthesis of VOCs extremely difficult and complex. Hence, the volatile compounds are not usually used for chemotaxonomic purposes. One study that explores VOCs on a germplasm level is by Yang et al. (2009), who evaluated the composition and concentration of volatiles in berries of 42 grape cultivars belonging to seven genotypic groups. Based on the aromatic profile and PCA analysis, the authors divided cultivars into three groups: 1. *V. labrusca* and its hybrids with *V. amurensis* or *V. vinifera*; 2*. V. vinifera* with muscat aroma; 3. others, including *V. vinifera* without muscat aroma plus *V. amurensis*, and hybrids between *V. vinifera* and *V. thunberghii* or *V. amurensis*. The authors also observed that quantitative variations of VOCs were influenced by the growing season, but the qualitative volatile composition of the cultivars was consistent.

Since research of VOCs on *V. vinifera*’s germplasm level is rare, the aim of this study is to analyze volatile profiles of red varieties from different GEN-GEO groups and to determine the differences between these groups based on the analyzed volatile profiles. Furthermore, using discriminant analysis, we wanted to determine the individual compounds contributing to the differences between GEN-GEO groups. This research included 50 grapevine varieties with different genetic and geographic origins. As mentioned, the GEN-GEO groups are based on the work of Laucou et al. (2018).

## Materials and methods

### Grape samples

Grape samples were collected during the 2019 growing season at proximity of full ripeness. During the season, maturity controls were performed by measuring the sugar content and visually by checking the seed color. Samples were collected when the sugar content stopped increasing and the seeds were brown in color. The samples were collected from 21 August to 30 August. Only true-to-type accessions were chosen to represent three GEN-GEO groups and five countries of origin, and were collected in a single collection in the INRAE Grape Germplasm Repository ‘Domaine de Vassal’. The grapevine varieties are grown on sandy soil on their own roots.

For each grape variety, five clusters were randomly chosen from three vines. Berries with attached pedicels were removed from the clusters using small scissors. One hundred and fifty berries were randomly chosen and divided into three batches of 50 berries each. Each batch was considered as one replication, resulting in three replications for each grape variety. The same replications were used for the analysis of free VOCs. Until analysis, the samples were stored at −20°C. The remainder of berries (~300) were removed from the clusters, divided into three uniform batches, and manually crushed to obtain juice for analysis of basic parameters such as total soluble solids, titratable acidity, and pH value. The basic parameters were measured according to the methods of the International Organization of Vine and Wine (OIV, 2019). The results of the analysis of basic parameters have been published by Šikuten et al. (2021b) as Supplementary material.

The selection of varieties was based on their genetic and geographic origin. The groups are as follows: C2 (10 grape varieties from France, 10 grape varieties from Italy), C7 (10 grape varieties from Croatia), and C8 (10 grape varieties from Spain, 10 grape varieties from Portugal). The country of origin was confirmed by the *Vitis* International Variety Catalogue (*V*IVC). The list of varieties, their country of origin, and GEN-GEO groups are presented in Supplementary Table 1.

### Analysis of volatile organic compounds

#### SPME-arrow extraction of free VOCs

For the analysis of VOCs, three batches of 50 berries (representing three replications) for each grape variety were used. The grape skins were manually removed from the frozen berries and freeze-dried. To obtain powder, the skins were ground using a MiniG Mill (SPEX Sample Prep, Meutchen, United States) and were stored at −20°C until analysis.

SPME-Arrow extraction was carried out based on the method described by Šikuten et al. (2021a). Briefly, SPME-Arrow extraction was conducted using an RSH Triplus autosampler (Thermo Fisher Scientific Inc., Brookfield, United States). Each sample weight of 100 mg was placed in a 20 ml headspace screw-top vial with a cap consisting of a PTFE/silicone septum.

The sorption conditions were as follows: the sample was incubated at 60°C for 20 min, and then the SPME-Arrow fiber DVB/CWR/PDMS (120 μm × 20 mm; Thermo Fisher Scientific Inc., Brookfield, United States) was exposed for 49 min. Then, the fiber was inserted into a GC injector port operating in splitless mode and desorbed at 250°C for 10 min.

#### GC–MS analysis

**Figure 1:**
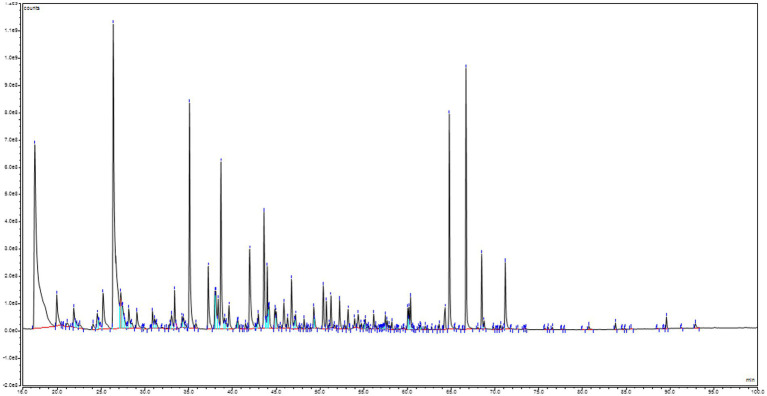
Typical GC–MS chromatogram of analyzed samples.

Separation and detection of the analytes was carried out by TRACE™ 1300 Gas Cromatographer coupled with ISQ 7000 TriPlus quadrupole mass spectrometer (Thermo Fisher Scientific Inc., Bartlesville, OK, United States) equipped with TG-WAXMS A capillary column (60 m × 0.25 mm × 0.25 μm film thickness; Thermo Fisher Scientific Inc., Bartlesville, OK, United States). The volatile compounds injected into the inlet were delivered to the column in splitless mode, and helium was used as a carrier gas at a constant flow rate of 1 ml/min. The oven temperature program was as follows: the initial temperature of 40°C was maintained for 5 min, increased by 2°C every minute until the temperature reached 210°C, and held for 10 min. The MS spectra was recorded in the electron impact ionization mode (EI) at an ionization energy of 70 eV. The mass spectrometer performed in full scan mode in the range of 30–300 *m/z*. The obtained data was processed using Chromeleon™ Data System (Thermo Fisher Scientific Inc., Bartlesville, OK, United States). Identification of volatile compounds was achieved by comparing the recorded mass spectrum with the data available in the Wiley Registry 12th Edition/NIST Spectral Library. The Retention Index (RI) was calculated using alkane standards C_8_–C_20_ (Sigma Aldrich, St. Louis, United States) according to the equation in Song et al. (2019) and compared with results previously reported in the literature (Babushok and Zenkevich, 2009; Babushok et al., 2011). The results are presented in Supplementary Table 2. All the results are expressed as absolute peak areas (×10^6^). Figure 1 represents a typical chromatogram.

### Statistical analysis

The individual VOCs were analyzed using one-way ANOVA, and the differences between given means for countries and GEN-GEO groups were evaluated by Duncan’s multiple range test at a confidence level of 95% (*p* < 0.05). The data reported in all the tables are the average triplicate observations. Discrimination among five groups of varieties based on country of origin and among three different GEN-GEO groups was performed by discriminant analysis (DA) stepwise forward model using the average grape skin volatile profiles of varieties to define multivariate difference among these groups, as well as to define the contribution of VOCs in discrimination. Statistical analysis was carried out using XLSTAT (Addinsoft, 2021, New York, United States).

## Results

### Volatile profile

In Supplementary Tables 3, 4 are given mean values of analyzed volatile organic compounds (VOCs) for all varieties, countries, and GEN-GEO groups. In total we analyzed 119 volatile compounds, among which are 50 sesquiterpenes, 28 alcohols, 16 aldehydes, 8 acids, 8 monoterpenes, 3 ketones, 3 lactones, 2 esters, and 2 C_13_-norisoprenoids.

#### Carbonyls, alcohols, acids, and esters

Carbonyl compounds (aldehydes and ketones) represent the most abundant group of VOCs, representing almost 50% of total VOCs. The most abundant compounds were (*E*)-2-hexenal, representing 60.83% of total carbonyls, and hexanal, representing 27.08% of total carbonyls. The compounds nonanal, benzaldehyde, and 2,4-hexadienal were found in higher abundance, while other compounds were found in low abundance or were detected in a small number of varieties (for example (*E,Z*)-2,6-nonadienal and phenylacetaldehyde). Varieties with the highest abundance of total carbonyls were Dobričić, Cahors, and Trepat. These varieties also had the highest abundance of (*E*)-2-hexenal and hexanal. On the other hand, the lowest abundance of total carbonyls were found in varieties Vranac, Icod do Vinao (Listan negro), and Rudežuša. In addition, these varieties had the lowest abundance of (*E*)-2-hexenal and hexanal. The C_9_ compound 2-nonenal was found in all varieties, except in Mencía and Rudežuša. In the case of (*E,Z*)-2,6-nonadienal, it was identified in seven varieties, namely Ancellotta, Servanin, Petit Verdot, Tinto Cão, Cabernet franc, Montepulciano, and Barbera. The GEN-GEO groups a had similar abundance of total carbonyl compounds. Similar to the varieties, the most abundant compounds were (*E*)-2-hexenal and hexanal, but the GEN-GEO groups did not differ significantly in the content of these compounds. However, they did differ in the abundance of compounds found in small amounts, such as 4-pentenal, decanal, benzaldehyde, and 6-methyl-5-heptene-2-one. Similar to the GEN-GEO groups, there were no significant differences in the abundance of total carbonyl compounds, (*E*)-2-hexenal, and hexanal based on country of origin.

Alcohols were the second most abundant class of compounds after carbonyls. The most abundant alcohol compounds were (*E*)-2-hexen-1-ol, representing 28.87% of total alcohols, followed by 1-hexanol, representing 24.6% of total alcohols, and benzyl alcohol, representing 15.62% of total alcohols. Other alcohol compounds found in higher abundance were (*Z*)-3-hexen-1-ol, isoamyl alcohol, and phenyl ethanol. Alcohol compounds represented in small abundance or detected in a small number of varieties included 1-nonanol and 2-nonanol. The varieties with the highest abundance of total alcohols were Petit Verdot, Tannat, and Touriga nacional. (*E*)-2-hexen-1-ol, as the most abundant alcohol, had the highest abundance in varieties Petit Verdot and Tinto Cão, while Vranac had a considerably lower abundance than the other varieties, followed by Garnacha. A considerably higher abundance of 1-hexanol was found in the variety Alvarelhão. The Tannat variety had the next highest abundance of 1-hexanol, at one times lower than Alvarelhão. The results for the GEN-GEO groups are similar to those of varieties, with (*E*)-2-hexen-1-ol and 1-hexanol as the most abundant compounds. The content of the above-mentioned compounds was similar for groups C2 and C8, while group C7 had a significantly lower abundance. The same results were found for total abundance of alcohols. When looking at countries of origin, the smallest abundance of total alcohols was found in Croatian varieties, followed by a similar content in Spanish and Italian varieties. The highest content of total alcohols was found in French and Portuguese varieties. It is a similar situation with the most abundant compounds, (*E*)-2-hexen-1-ol and 1-hexanol.

Only two compounds belonging to esters were found, ethyl hexanoate and ethyl octanoate. Ethyl hexanoate was only found in two Spanish varieties, Sumoll Tinto and Carignan. Ethyl octanoate was identified in all varieties, except Barbera, while Mancens had the highest abundance.

Acids are another class of volatile compounds identified in the analyzed grapevine varieties. This class of compounds represented only 4% of total VOCs, with eight compounds identified. The most abundant acid was hexanoic acid, representing 62.49% of total acids, followed by (*E*)-2-hexenoic acid, representing 22.34% of total acids. Other acids found in higher abundance were nonanoic and benzoic acids. The variety with a significantly higher abundance of total acids was Trepat, followed by Mourisco tinto and Barbera. These varieties also had the highest abundance of hexanoic and (*E*)-2-hexenoic acid. The smallest abundance of total acids was found in varieties Lasina and Ninčuša, which also had the smallest abundance of the most abundant acids. The results for the GEN-GEO groups followed the results of the individual varieties. Group C7 had the lowest abundance of total acids, hexanoic and (*E*)-2-hexanoic acid, while groups C2 and C8 did not differ in the abundance of mentioned parameters. In the context of countries of origin, Croatian varieties had the lowest abundance of all identified acids and total acids, while other countries had similar abundance.

#### Terpenoids

In our samples, the representatives of the terpenoid family are monoterpenes, sesquiterpenes, and C_13_-norisoprenoids. Only eight monoterpene compounds were identified in analyzed samples and in relatively small quantities. The variety with a significantly higher abundance of monoterpenes was Dolcetto, followed by Terrano and Baga, while the lowest abundance was found in varieties Crljenak kaštelanski, Sušćan, and Ninčuša. The most abundant monoterpene was *β*-pinene, followed by 2-pinen-4-one and *β*-ocimene. Among monoterpene alcohols, only linalool was identified but in small quantities. Regarding the GEN-GEO groups, the C7 group had the lowest abundance of total monoterpenes, while the C2 and C8 groups had similar abundance. Similar results were shown for countries of origin. Croatian varieties mostly differed from the others by having a low abundance of analyzed monoterpenes. Varieties from other countries differed slightly in the abundance of monoterpenes.

In analyzed varieties, sesquiterpenes represented the third most abundant group of VOCs, comprising 21.6% of total VOCs. The most abundant compound was ylangene, followed by *β*-copaene and *β*-burbonene. Some of the compounds were found in only a few varieties, like isospathulenol, (*Z*)-*β*-farnesene, or *α*-farnesene. The varieties with the highest abundance of sesquiterpenes were Dolcetto and Baga, while the lowest abundance was recorded for Vranac, Mancin, and Tinto Cão. The highest abundance of ylangene was recorded in Trepat, Baga, and Terrano, while the lowest abundance was recorded in Montepulciano, Manseng, and Ninčuša. The GEN-GEO groups did not differ significantly in the abundance of total sesquiterpenes. The content of ylangene, the most abundant compound, was significantly higher in group C8, while the abundance in groups C2 and C7 was similar. When looking at the countries of origin, the abundance of total sesquiterpenes did differ significantly. The highest abundance was recorded for Italian and Portuguese varieties, followed by Spanish varieties. Croatian and French varieties had the lowest abundance of sesquiterpenes, which is in accordance with the results presented for varieties and GEN-GEO groups. The content of ylangene also follows the results presented for GEN-GEO groups. Hence, the Croatian and French varieties showed the lowest abundance, while the highest abundance was recorded for Spanish and Portuguese varieties, followed by Italian varieties.

In analyzed varieties, only two C_13_-norisoprenoids were detected, (*E*)-β-ionone and TDN. The varieties with a considerably higher abundance of C_13_-norisoprenoids included Dolcetto and Tinto Cão, while the lowest abundance was recorded for Mancens and Soić. Among the GEN-GEO groups, group C7 had the lowest abundance of analyzed norisoprenoids. Groups C2 and C8 had a similar content of analyzed norisoprenoids. The results based on countries of origin followed those reported for GEN-GEO groups. Hence, the Croatian varieties had the lowest abundance of analyzed norisoprenoids, and varieties from other countries did not differ in the content of norisoprenoids.

### Discriminant analysis

**Figure 2:**
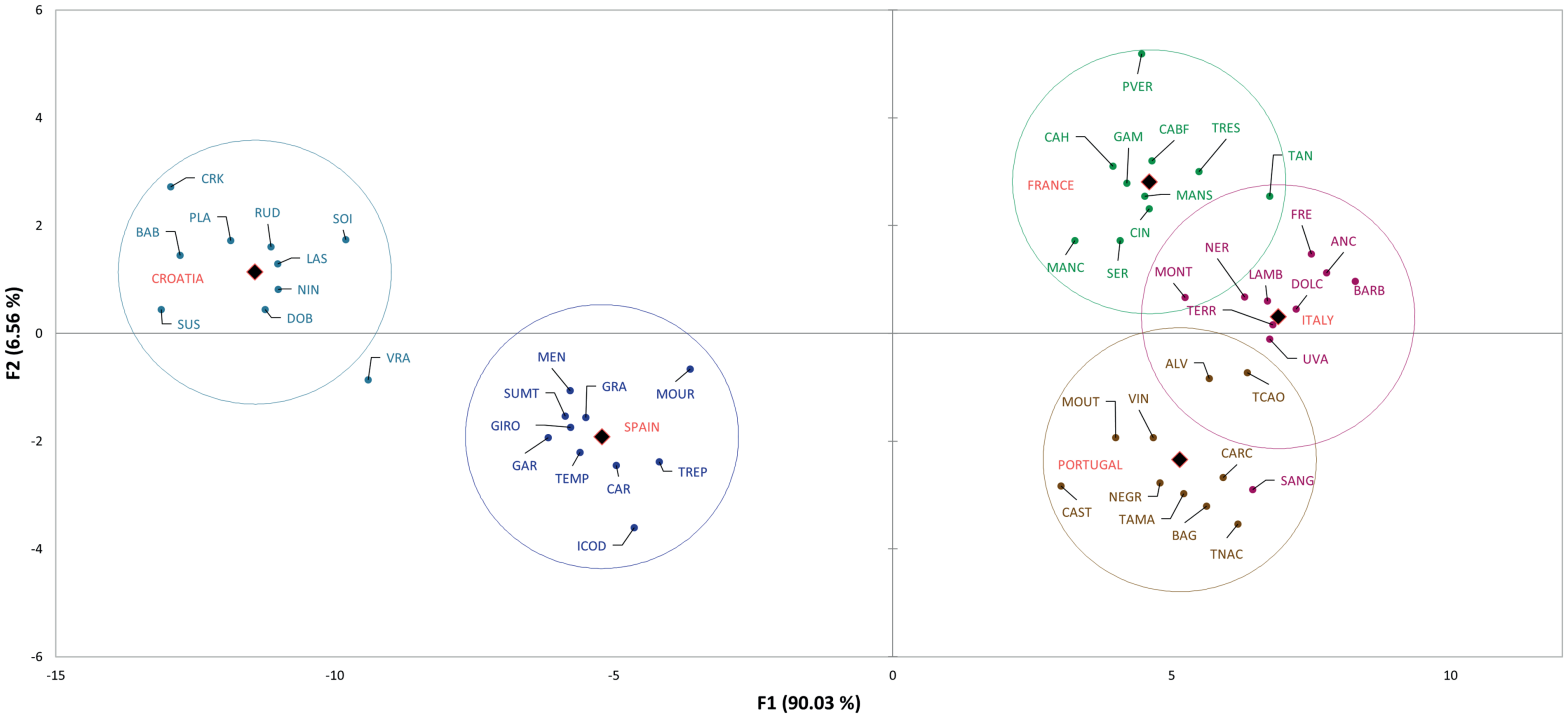
The scatter plot of discriminant analysis representing cultivars from different countries.

The discriminant analysis (DA) of varieties based on their country of origin is presented in Figure 2, which shows the distribution of the varieties in the space defined with the first two canonical factors. The factors explained 96.59% of variability (F1 90.03%, F2 6.56%). In Supplementary Table 5, the correlations of variables/factors are shown, which represent the compounds contributing the most to the discrimination. As presented in Figure 1, the countries are distinctly separated based on their volatile profile. Croatian and Spanish varieties are distinctly separated and are located in the first and third quadrant, respectively. It can be seen that Croatian varieties are discriminated by the compounds benzoic acid, isoamyl alcohol, heptanal, hexanal, and (*S,R*)-2,3-butanediol. Spanish varieties are discriminated by *exo*-2-hydroxycineole, pentanoic acid, *γ*-undelactone, and isoamyl alcohol. French and Italian varieties are located in the second quadrant, but they are clearly separated. These varieties are differentiated mostly by alcohols, chiefly 3-methoxy-1-butanol, (*Z*)-2-penten-1-ol, (*E*)-2-hexen-1-ol, and 2-nonanol. Furthermore, two other compounds had a high correlation with certain varieties, namely 4-pentenal in French varieties, and nonanoic acid in Italian varieties. Portuguese varieties were located in the fourth quadrant and were differentiated by the presence of linalool, (*E*)-2-hexenoic acid, ylangene, and 1-butoxy-2-propanol.

**Figure 3:**
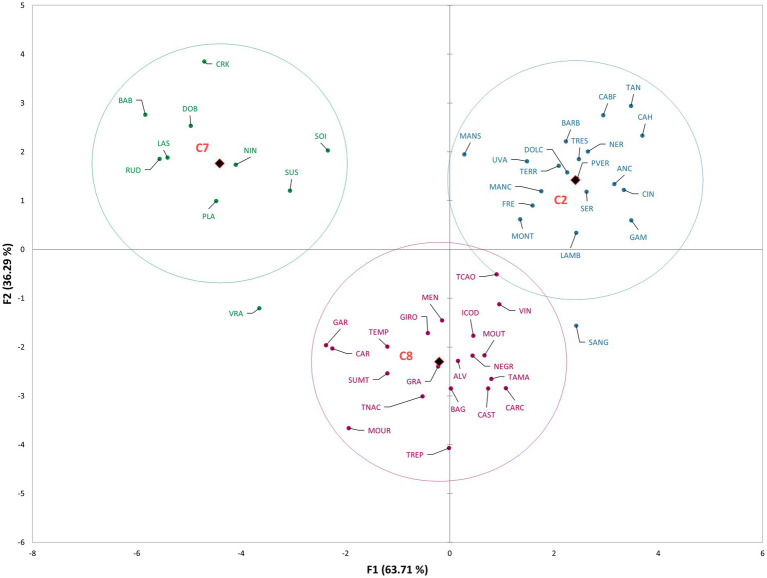
The scatter plot of discriminant analysis representing cultivars from different GEN-GEO groups.

Figure 3 represents the results of DA for GEN-GEO groups, that is, the distribution of varieties in the space defined with the first two canonical factors, explaining 100% of variability (F1 63.71%, F2 36.29%). In Supplementary Table 3 the variable/factor correlations are presented. Similar to the groups based on countries of origin, the GEN-GEO groups were also distinctly separated. Group C7 located in the first quadrant, containing Croatian varieties, was differentiated by the presence of compounds benzoic acid, heptanal, (*E*)-*α*-bergamontene, and both (*S,R*)- and (*R,R*)-2,3-butanediol. The second quadrant contains group C2, discriminated by the presence of compounds (*E*)-2-hexen-1-ol, 2,4-dimethyl-3-pentanol, (*E,E*)-2,4-hexadienal, and (*E*)-2-hexenal. Group C2 consists of French and Italian varieties, with high correlations of 4-pentenal and nonanoic acid. Group C8 is located in third and fourth quadrant, near the y axis, and was discriminated by the presence of compounds *exo*-2-hydroxycineole, isoamyl alcohol, (*E*)-*β*-ionone, linalool, (*E*)-2-hexenoic acid, and phenyl ethanol.

## Discussion

Fifty grapevine varieties, representing different GEN-GEO groups and countries of origin, varied greatly in their volatile profile. VOCs in grapes are represented by different groups of compounds including aldehydes, alcohols, esters, and acids. Although these compounds are mostly produced during fermentation by yeast metabolism, many compounds originate directly from grapes. In our samples, the most abundant are C_6_ aldehydes; however, some of the most powerful aroma compounds have nine carbon atoms, such as 2-nonenal or (*E,Z*)-2,6-nonadienal, contributing, like all aldehydes, to green and herbaceous aromas (Ferreira and Lopez, 2019). Furthermore, their content during ripening can be extremely low (Zhu et al., 2012), which can also be seen in our results. Alcohols, like aldehydes, contribute to green and herbaceous aromas. Besides being important aroma contributors, alcohols could also be used as varietal markers. In a study on C_6_ alcohols, Oliveira J. M. et al. (2006) showed that 1-hexanol, (*E*)-3-hexanol, and (*Z*)-3-hexanol could be used in discrimination of varieties. Regarding esters, they are powerful odorants, with a very low odor threshold (Pineau et al., 2009). In the analyzed samples, only two esters were identified, belonging to the group of ethyl esters: ethyl hexanoate contributed to the apple-like and aniseed aromas, and ethyl octanoate contributed to the sour apple aroma (Saerens et al., 2010). Most of the esters are produced during fermentation by yeast metabolism, and the production is dependent on the presence of precursors in the must (Saerens et al., 2010; Dennis et al., 2012; Boss et al., 2015). Furthermore, in a study on Cabernet Sauvignon, the esters were identified from early developmental stages of grape berries. However, their concentrations significantly dropped at vérasion (Kalua and Boss, 2009). Thus, this could be an explanation as to why only two compounds were identified. All these classes of compounds are mutually connected through biosynthetic pathways and can be transformed into each other. In grapes, C_6_ and C_9_ alcohols and aldehydes are products of lipoxygenase pathway (Lin et al., 2019). In this pathway, the fatty acids are oxidized by lipoxygenases (LOX) and modified by hyperoxide lyase to form aldehydes (Dunlevy et al., 2009). The most abundant acids in our samples were (*E*)-2-hexenoic and hexanoic acids, which through the above-mentioned modifications can yield aldehydes (*E*)-2-hexenal and hexanal, the most abundant aldehydes. The produced aldehydes can be further metabolized by alcohol dehydrogenase (ADH) to form corresponding alcohols (Schwab et al., 2008), (*E*)-2-hexen-1-ol and 1-hexanol. The production of ethyl esters is dependent on the concentration of fatty acid precursors (Saerens et al., 2010), which in our samples were hexanoic and octanoic acids. Although hexanoic acid was the most abundant acid, it did not yield esters, except in two varieties. On the other hand, octanoic acid was not identified in grape samples, but the corresponding ester, ethyl octanoate, was identified. Thus, it could be hypothesized that the small quantities produced were transformed into esters.

Terpenoids are the most extensively studied group of VOCs in *Vitis vinifera* grapes and are an extremely diverse and abundant group (Yu and Utsumi, 2009; Gonzalez-Barreiro et al., 2015). The major representatives are monoterpenes, sesquiterpenes and norisoprenoids, which are all responsible for fruity (citric) and floral aromas of grapes and wines (Gonzalez-Barreiro et al., 2015; Lin et al., 2019). As presented, there were only 8 monoterpene compounds identified in analyzed samples. This is not surprising since red varieties are not characterized by high levels of terpenes (Hernandez-Orte et al., 2015; Yuan and Qian, 2016; Luo et al., 2019). Furthermore, the levels of bound monoterpenes, which were not included in this research, are usually much higher than levels of free monoterpenes (Li et al., 2017; Yue et al., 2020). Sesquiterpenes in grapes and wines have received less attention due to their lower volatility and detection threshold (Lin et al., 2019). In total we identified 50 sesquiterpene compounds, which makes sesquiterpenes the most numerous class of VOCs. The most known compound is rotundone, which was not identified in our samples. Regarding other sesquiterpenes, May and Wust (2012) showed that grapes emit numerous sesquiterpene hydrocarbons and the sesquiterpene profile depends on grape variety and developmental stage. Norisoprenoids are another class of terpenoid compounds found in grapes and wines. Among these carotenoid-derived compounds, C_13_-norisoprenoids are the most widespread (Winterhalter and Rouseff, 2002). In analyzed samples only two compounds belonging to norisoprenoids were identified and in small quantities. However, other research on red varieties reported the presence of other norisoprenoids, such as *β*-damascenone or *α*-ionone (Oliveira C. et al., 2006; Bindon et al., 2007; Yuan and Qian, 2016). What we did find in accordance with these researches are the low abundances of identified compounds. Since the majority of norisoprenoids, like monoterpenes, are found in non-volatile bound form (Lin et al., 2019; Mele et al., 2021), this could be potential explanation for detecting only two compounds in relatively small abundance.

Research on germplasm level that explore volatiles in grapevine varieties are scarce and do not include genetic and geographic origin. In our work when talking about geographic origin, we are referring to it in the sense of where these varieties are considered to be native, not in the sense of terroir or winegrowing regions. Furthermore, the samples were collected from a single location with the aim of minimalizing the effect of different environmental factors and rootstocks (Olarte Mantilla et al., 2018; Carrasco-Quiroz et al., 2020).

The discriminant analysis clearly separated the groups based on the country of origin and GEN-GEO groups. The mean values of compounds, above-mentioned to discriminate the groups, are the highest for the country of origin that they discriminate (Supplementary Tables 3, 4). However, when looking at the whole volatile profile, these compounds are found in relatively small abundance or in just few varieties. For example, *γ*-undelactone was identified in only four varieties, Rudezusa (Croatia), Uva rara (Italy), Graciano and Trepat (Spain). Nevertheless, they contribute to the discrimination and show that these small quantities identified are important varietal characteristics, that is characteristics defined by geographical origin. Alongside these compounds, the overall volatile profile also contributed to discrimination, especially to the French, Italian and Portuguese varieties, which are closely located on the scatter plot. These groups of varieties, as can be seen from correlations and mean values, are characterized by high content of alcohols, carbonyls, and sesquiterpenes. Similar results were obtained for GEN-GEO groups, and figures show similar position on scatter plots. GEO groups, like countries of origin, were discriminated by the compounds found in high quantities for the group that they discriminate. However, in the overall volatile profile, these compounds represent a small proportion. Furthermore, GEO groups are also discriminated by their overall volatile profile. Again, based on the correlations and mean values, it can be seen that C2 group contains higher abundance of carbonyl compounds and alcohols, while C8 group contains higher abundance of sesquiterpenes and acids. Group C7 is not characterized by high quantities of VOCs, except the compounds that discriminate it. Regarding the C8 group and its position on the scatter plot, it is interesting that the varieties separated within group, with Spanish varieties near y axis in the third quadrant, and Portuguese varieties near y axis in the fourth quadrant. That clear separation within group was not visible in group C2, containing French and Italian varieties. In the context of winemaking, even the small changes or differences in volatile profiles can have an impact on the sensory properties of wine (Ilc et al., 2016; Ferreira and Lopez, 2019).

On both figures representing DA results, varieties Vranac and Sangiovese singled out from their groups. In Figure 2 Sangiovese was located in the group containing Portuguese varieties and within the group near the varieties Carcajolo and Touriga nacional. Comparing the mean values of these three varieties, as well as the mean values of all Portuguese varieties, Sangiovese indeed has similar profile to Portuguese varieties. Although Sangiovese was not included by DA in group C8, on scatter plot is located more closely to group C8 and Portuguese varieties, than to group C2 containing all other Italian varieties. Vranac on the other hand was not placed near any group. The reason is probably its poor volatile profile, compared even to the Croatian varieties, which in general had low quantities of VOCs.

The volatile profiles of grape varieties are complex and include a large number of compounds. This research gives insight into the volatile profiles of red grape varieties with different genetic and geographic background. The most abundant compounds were carbonyls, while sesquiterpenes were the most numerous. Discriminant analysis clearly separated both countries of origin and GEN-GEO groups based on their volatile profile, with all classes of compounds contributing to the discrimination.

## Data availability statement

The original contributions presented in the study are included in the article/Supplementary material, further inquiries can be directed to the corresponding author.

## Author contributions

IŠ: conceptualization, investigation, and writing–original draft. PŠ: formal analysis and investigation. IT: methodology. CM: resources. JK: writing–review and editing. TL: resources, writing–review and editing. EM: funding acquisition. DP: conceptualization, formal analysis, and writing–review and editing, supervision. All authors contributed to the article and approved the submitted version.

## Funding

This work was supported by Centre of Excellence for Biodiversity and Molecular Plant Breeding (CoE Crop-BioDiv KK.01.1.1.01.005).

## Conflict of interest

The authors declare that the research was conducted in the absence of any commercial or financial relationships that could be construed as a potential conflict of interest.

## Publisher’s note

All claims expressed in this article are solely those of the authors and do not necessarily represent those of their affiliated organizations, or those of the publisher, the editors and the reviewers. Any product that may be evaluated in this article, or claim that may be made by its manufacturer, is not guaranteed or endorsed by the publisher.
