# Supplementary material for: Discrimination of genetic and geographical groups of grape varieties (Vitis vinifera L.) based on their volatile organic compounds
Source: Front Plant Sci. 2022 Oct 20;13:942148. doi: 10.3389/fpls.2022.942148 (PMC9634546; doi:10.3389/fpls.2022.942148)
Supplement: Supplementary file 1 [file Table_1.DOCX]

Supplemental table 1. List of cultivars and their country of origin and GEO group

|  | Cultivar | Country of origin | GEO group |
| --- | --- | --- | --- |
| 1. | Babica | Croatia | C7 |
| 2. | Tribidrag | Croatia | C7 |
| 2. | Dobricic | Croatia | C7 |
| 4. | Lasina | Croatia | C7 |
| 5. | Nincusa | Croatia | C7 |
| 6. | Plavina | Croatia | C7 |
| 7. | Rudezusa | Croatia | C7 |
| 8. | Soic | Croatia | C7 |
| 9. | Suscan | Croatia | C7 |
| 10. | Vranac | Balkan | C7 |
| 11. | Carignan | Spain | C8 |
| 12. | Garnacha | Spain | C8 |
| 13. | Mancens | Spain | C8 |
| 14. | Graciano | Spain | C8 |
| 15. | Icod do vinao | Spain | C8 |
| 16. | Mencia | Spain | C8 |
| 17. | Mourvedre | Spain | C8 |
| 18. | Sumoll tinto | Spain | C8 |
| 19. | Tempranillo | Spain | C8 |
| 20. | Trepat | Spain | C8 |
| 21. | Cabernet franc | France | C2 |
| 22. | Cahours | France | C2 |
| 23. | Cinsaut | France | C2 |
| 24. | Gamay | France | C2 |
| 25. | Mancin | France | C2 |
| 26. | Manseng noir | France | C2 |
| 27. | Petit Verdot | France | C2 |
| 28. | Servanin | France | C2 |
| 29. | Tannat | France | C2 |
| 30. | Tressot | France | C2 |
| 31. | Ancellotta | Italy | C2 |
| 32. | Barbera | Italy | C2 |
| 33. | Dolcetto | Italy | C2 |
| 34. | Freisa | Italy | C2 |
| 35. | Lambrusco Maestri | Italy | C2 |
| 36. | Montepulciano | Italy | C2 |
| 37. | Nerello Cappuccio | Italy | C2 |
| 38. | Sangiovese | Italy | C2 |
| 39. | Terrano | Italy | C2 |
| 40. | Uva rara | Italy | C2 |
| 41. | Alverlhao | Portugal | C8 |
| 42. | Baga | Portugal | C8 |
| 43. | Carcajolo | Portugal | C8 |
| 44. | Castelao | Portugal | C8 |
| 45. | Mourisco tinto | Portugal | C8 |
| 46. | Negra mole | Portugal | C8 |
| 47. | Tinta Amarela | Portugal | C8 |
| 48. | Tinto Cao | Portugal | C8 |
| 49. | Touriga nacional | Portugal | C8 |
| 50. | Vinhao | Portugal | C8 |
